# Expression of lipocortins in human bronchial epithelial cells: effects of IL-1β , TNF-α, LPS and dexamethasone

**DOI:** 10.1155/S0962935196000300

**Published:** 1996-06

**Authors:** M. M. Verheggen, H. I. M. de Bont, P. W. C. Adriaansen-Soeting, B. J. A. Goense, C. J. A. M. Tak, H. C. Hoogsteden, P. Th. W. van Hal, M. A. Versnel

**Affiliations:** 1 Department of Immunology Erasmus University and Academic Hospital Dijkzigt Rotterdam The Netherlands; 2 Department of pulmonary Medicine Erasmus University and Academic Hospital Dijkzigt Rotterdam The Netherlands; 3 Department of pharmacology Erasmus University and Academic Hospital Dijkzigt Rotterdam The Netherlands

## Abstract

In this study, we investigated the expression of lipocortin I and II (annexin I and I in the human bronchial epithelium, both *in vivo* and *in vitro*. A clear expression of lipocortin I and II protein was found in the epithelium in sections of bronchial tissue. In cultured human bronchial epithelial cells we demonstrated the expression of lipocortin I and II mRNA and protein using Northern blotting, FACScan analysis and ELISA. No induction of lipocortin I or II mRNA or protein was observed after incubation with dexamethasone. Stimulation of bronchial epithelial cells with IL-1β, TNF-α or LPS for 24 h did not affect the lipocortin I or II mRNA or protein expression, although PGE_2_ and 6-keto-PGF_1α_ production was significantly increased. This IL-1β- and LPS-mediated increase in eicosanoids could be reduced by dexamethasone, but was not accompanied by an increase in lipocortin I or II expression. In human bronchial epithelial cells this particular glucocorticoid action is not mediated through lipocortin I or II induction.


Mediators of Inflammation, 5, 210–217 (1996)

### Expression of lipocortins in human bronchial epithelial cells: effects of IL-1β, TNF-α, LPS and dexamethasone

M. M. Verheggen,[1,2] H. I. M. de Bont,[1]

P. W. C. Adriaansen-Soeting,[1] B. J. A. Goense,[1]

C. J. A. M. Tak,[3] H. C. Hoogsteden,[2]

P. Th. W. van Hal[1,2] and M. A. Versnel[1,CA]

Departments of [1]Immunology, [2]Pulmonary Medicine and [3]Pharmacology, Erasmus University and Academic Hospital Dijkzigt, Rotterdam, The Netherlands

[CA]Corresponding Author

**In this study, we investigated the expression of lipocortin I and II (annexin I and II) in the human bronchial epithelium, both *in vivo* and *in vitro*. A clear expression of lipocortin I and II protein was found in the epithelium in sections of bronchial tissue. In cultured human bronchial epithelial cells we demonstrated the expression of lipocortin I and II mRNA and protein using Northern blotting, FACScan analysis and ELISA. No induction of lipocortin I or II mRNA or protein was observed after incubation with dexamethasone. Stimulation of bronchial epithelial cells with IL-1β, TNF-α or LPS for 24 h did not affect the lipocortin I or II mRNA or protein expression, although $PGE_2$ and 6-keto-$PGF_{1\alpha}$ production was significantly increased. This IL-1β- and LPS-mediated increase in eicosanoids could be reduced by dexamethasone, but was not accompanied by an increase in lipocortin I or II expression. In human bronchial epithelial cells this particular glucocorticoid action is not mediated through lipocortin I or II induction.**

**Key words**: Bronchial epithelial cells, Eicosanoids, Glucocorticoids, Lipocortins

### Introduction

The bronchial epithelium is considered to play an important role in initiating and perpetuating inflammatory and immunological reactions by production of a variety of inflammatory mediators. Therefore, it is thought that human bronchial epithelial cells (HBEC) may play a role in inflammatory pulmonary diseases such as asthma. Upon *in vitro* stimulation with inflammatory agents such as interleukin-1β (IL-1β), tumour necrosis factor-α (TNF-α) and lipopolysaccharide (LPS), HBEC are able to produce several cytokines, such as IL-1, interleukin-6 (IL-6), interleukin-8 (IL-8), granulocyte-macrophage colony-stimulating factor (GM-CSF) and monocyte chemoattractant factor-1 (MCP-1).[1–3] Furthermore, HBEC exposed to various stimuli *in vitro* release several arachidonic acid metabolites, including 15-hydroxyeicosatetraenoic acid (15-HETE), prostaglandin $E_2$ ($PGE_2$) and 6-keto-prostaglandin $F_{1\alpha}$ (6-keto-$PGF_{1\alpha}$).[4,5] In addition to these pro-inflammatory properties, HBEC could also be involved in anti-inflammatory reactions through the production of potential anti-inflammatory proteins, e.g. lipocortins.

Lipocortin I and II (annexin I and II) are members of the annexin family of $Ca^{2+}$-dependent phospholipid-binding proteins.[6] Biological evidence suggests that at least some members of this family are glucocorticoid inducible proteins with anti-inflammatory properties.[7] It has been proposed that lipocortins I and II mediate part of the immunosuppressive activity of glucocorticoids by inhibiting phospholipase $A_2$ ($PLA_2$) activity, hereby preventing eicosanoid production.[8] In some recent studies, however, the induction of lipocortin I and II by glucocorticoids was not observed.[9–11] Other biological functions for lipocortins have also been reported, such as the regulation of cell differentiation and growth, and a role in the central nervous system and neuroendocrine system (reviewed in Reference 12).

Animal studies have suggested that the lung is a rich source of lipocortin I.[13–16] In the human lung, lipocortin synthesis has been described in blood leucocytes and alveolar macrophages.[17,18] In cultured human tracheal submucosal gland cells production of lipocortin-like proteins have been found.[19] However, to our knowledge, no data are available on the presence of lipocortins in HBEC.

The aim of this study was to investigate the expression of lipocortin I and II in HBEC and in the human bronchial epithelial cell line BEAS 2B, and to examine whether these potential anti-inflammatory proteins could be induced by glucocorticoids. Furthermore, we investigated the effect of inflammatory agents such as IL-1β, TNF-α and LPS on the lipocortin expression. Finally, we studied whether the effects of dexamethasone

© 1996 Rapid Science Publishers on the IL-1β- and LPS-stimulated production of $PGE_2$ and 6-keto-$PGF_{1\alpha}$ correlated with an induction of lipocortin I and II.

### Materials and Methods

*Isolation and culture conditions of HBEC:* Bronchi were obtained from patients undergoing surgery for lung cancer. Only bronchial tissue distant from the tumour and having a normal macroscopic appearance was used. Freshly removed tissue samples were collected in cold sterile HEPES buffered RPMI (GIBCO, Paisley, UK), supplemented with penicillin G sodium (100 U/ml; Gist-Procades, Delft, The Netherlands) and streptomycin sulphate (0.1 mg/ml; Biochrom KG, Berlin, Germany) for transport to the laboratory. Tissue samples were cut into pieces, washed with cold phosphate buffered saline (PBS), and incubated either overnight at 4°C or 1 h at 37°C in HEPES buffered RPMI containing 0.1% protease XIV (Sigma, St Louis, MO), penicillin G sodium (100 U/ml) and streptomycin sulphate (0.1 mg/ml). Subsequently, epithelial cells were gently scraped from the tissue samples, washed twice in culture medium and plated onto 35-mm dishes at a density of $2.5 \times 10^5$ cells/dish.

HBEC were cultured in a 1:1 mixture of Dulbecco's modified Eagle's medium and Ham's F12 (DMEM/F12) (GIBCO), supplemented with insulin (0.01 mg/ml; Sigma), hydrocortisone (1 μg/ml; Pharma Chemie, Haarlem, The Netherlands), transferrin (0.01 mg/ml; Behring Marburg, Germany), epidermal growth factor (EGF) (10 ng/ml; Collaborative Research Inc., Lexington, MA), foetal calf serum (FCS) (1%), $Na_2SeO_3$ (50 nM), glutamine (1 mM; JT Baker bv., Deventer, The Netherlands), penicillin G sodium and streptomycin sulphate (0.1 mg/ml). Cells were characterized as epithelial cells by immunofluorescence staining using a mouse monoclonal antibody directed against a number of human cytokeratins (CK-1; DAKOpatts, Glostrup, Denmark). At least 99% of the isolated cells stained positive for cytokeratin ($n = 5$).

*Human bronchial epithelial cell line:* BEAS 2B is a SV-40 transformed human bronchial epithelial cell line, which was kindly provided by Dr J. Lechner (Inhalation Toxicology Research Institute, Albuquerque, NM).[20] Cells were maintained in a keratinocyte growth medium containing bovine pituitary extract, EGF, penicillin G sodium and streptomycin sulphate (KGM; GIBCO).[21] Plastic cell culture plates (Falcon, Becton Dickinson, NJ) were precoated as described by Lechner *et al.* with a mixture of human fibronectin (10 μg/ml; Central Laboratory of the Blood Transfusion Service, Amsterdam, The Netherlands), collagen (Vitrogen 100, 30 μg/ml; Collagen Corp., Palo Alto, CA) and bovine serum albumin (BSA) (10 μg/ml; Boehringer, Mannheim, Germany) in PBS.[22] Before using the BEAS 2B cells for experiments, cells were cultured for one passage in DMEM/F12 with supplements, as described for HBEC cells.

*Immunofluorescence and immunoperoxidase stainings of cells and tissue:* Immunostainings were performed with a rabbit polyclonal antibody against lipocortin I, kindly provided by Dr R. B. Pepinsky and a mouse monoclonal antibody against lipocortin II (Oncogene Science Inc., Manhasset, NY). All antibody reagents were diluted in PBS containing (w/v) 0.5% BSA and 0.1% sodium azide. Normal mouse serum, normal rat serum and two subclass specific antibodies were used as negative controls.

*Cells.* For fluorescence activated cell scan (FACScan) experiments cells were detached with 0.02% EDTA, fixed for 40 min at room temperature (RT) with Permea-Fix Reagent (Ortho Diagnostic Systems, Beerse, Belgium) and washed for 10 min with PBS/BSA (0.5%). The cells were then incubated with anti-lipocortin I or II antibodies for 30 min on ice. After washing with PBS/BSA, cells were incubated for another 30 min with either fluorescein isothiocyanate (FITC)-conjugated goat anti-rabbit-IgG against lipocortin I or FITC-conjugated goat anti-mouse-IgG against lipocortin II. Subsequently, the cells were washed and staining was analysed and quantified using a FACScan flow cytometer (Becton Dickinson).

*Tissue.* Bronchi specimens for immunohistochemistry, from one female and two male non-asthmatic patients, were quickly frozen in liquid nitrogen and stored at –80°C. Six μm frozen bronchi sections were fixed in acetone for 1 min at RT and incubated with anti-lipocortin I or II antibodies for 60 min in a humidified chamber. The sections were then washed twice with PBS/Tween (0.1%) and incubated for 60 min with horseradish peroxidase (HRP)-conjugated swine anti-rabbit-IgG or HRP-conjugated rabbit anti-mouse-IgG for detection of lipocortin I and II, respectively (DAKOpatts). After three washing procedures with PBS/Tween, peroxidase activity was measured using diaminobenzidine tetrahydrochloride (Sigma) as substrate.

*Stimulation experiments with IL-1β, TNF-α and LPS in the presence of dexamethasone:* All experiments were performed on days 7 through 10 of primary culture of HBEC. Twenty-four h before treatment with cytokines and/or dexamethasone the medium was replaced by a basal medium consisting of DMEM/F12 without hydrocortisone or other supplements to prevent influence of endogenous steroids. IL-1β (20 ng/ml; UBI, Lake Placid, NY), TNF-α (20 ng/ml; UBI), LPS (10 or 100 μg/ml; Difco Laboratories, Detroit, MI) and/or dexamethasone ($10^{-6}$ M; Duchefa bv., Haarlem, The Netherlands) were added to the basal medium. After 24 h, supernatants were collected. Total RNA was isolated as described below. Supernatants and RNA were stored at −20°C and −80°C, respectively, until analysis.

*RNA isolation, Northern blot analysis and probes:* Total RNA was extracted as described previously and stored at −80°C until used.[23] Northern blotting and hybridization were performed as described previously.[24] The lipocortin I and II probes were a 1.3 and a 0.9 kb *Eco*RI fragment, respectively, kindly provided by Dr B. P. Wallner (Biogen Research Corp., Cambridge, MA). The glyceraldehyde-3-phosphate-dehydrogenase (GAPDH) probe was a 0.7 kb *Eco*RI-*Pst*I fragment.[25] The IL-8 probe was a 1.3 kb *Eco*RI fragment, kindly provided by Dr T. J. Stoof (Department of Dermatology, VU Hospital, Amsterdam, The Netherlands).

The intensity of the lipocortin I, lipocortin II, IL-8 and GAPDH mRNA signals on the autoradiograph were scanned with a handscanner (Colorscanner 2,[24] Highscreen, Würselen, Germany) at a resolution of 100 dots per inch. Computer software described by Koning *et al.* was used to analyse the intensity of the bands.[26] Values were expressed as the ratio of lipocortin to GAPDH mRNA intensity.

*Analysis of extracellular lipocortin I in culture supernatants:* Extracellular lipocortin I levels were kindly measured by Dr Susan F. Smith, using a competitive ELISA.[27]

*Production of arachidonic acid metabolites:* The arachidonic acid metabolites $PGE_2$ and 6-keto-$PGF_{1\alpha}$ were measured by radioimmunoassay as described previously.[28] Arachidonic acid metabolite release was normalized to total RNA content.

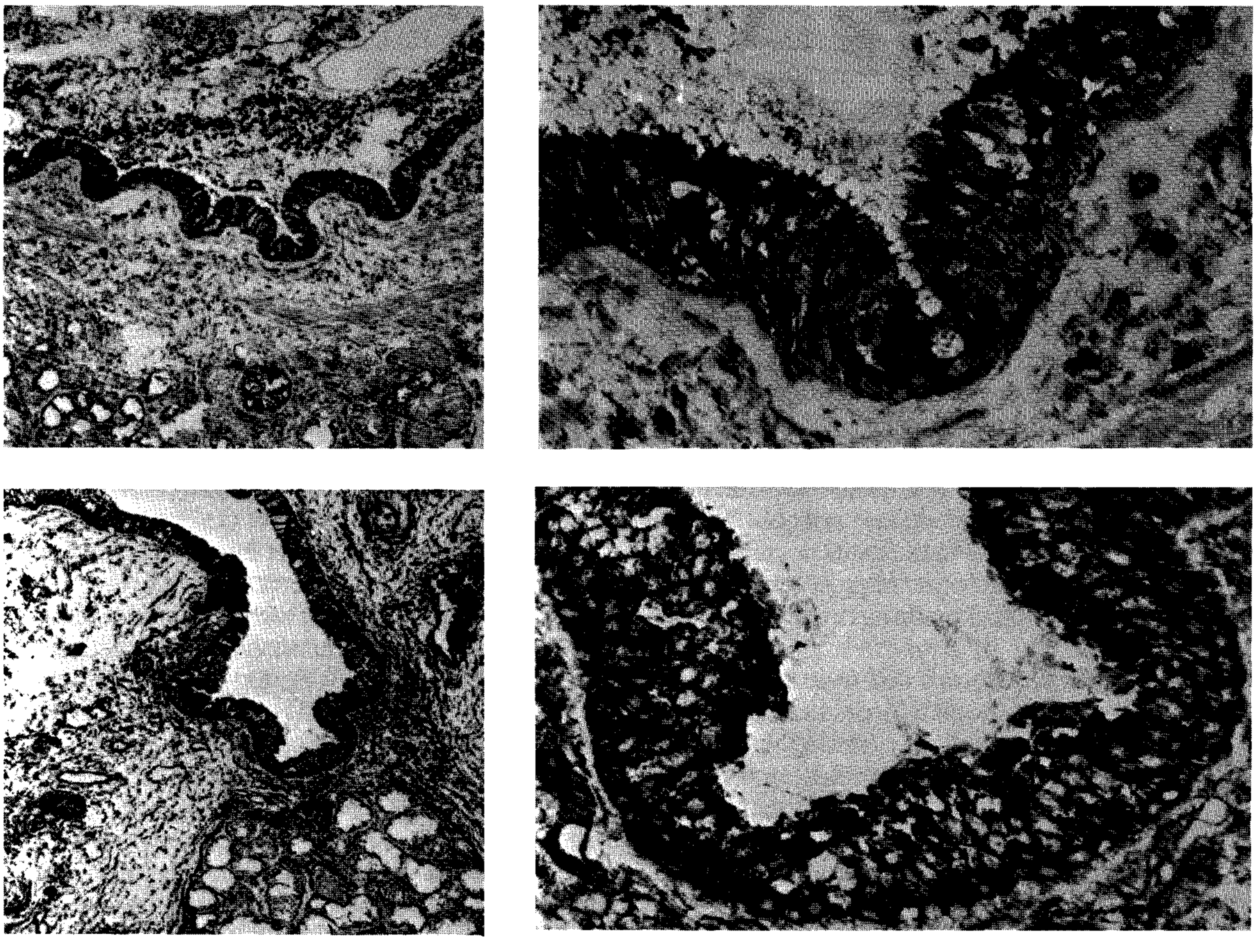

FIG. 1. Immunoperoxidase staining for lipocortin I (upper panel) and II (lower panel) of frozen sections of human bronchi. Left: magnification 6.3 ×. Right: magnification 40 ×.

*Statistical analysis:* The Mann–Whitney *U* test was used to assess significant differences in $PGE_2$ and 6-keto-$PGF_{1\alpha}$ production, and in lipocortin I and II mRNA expression in cell cultures under different conditions of incubation. A *p*-value of less than 0.05 was considered significant.

### Results

*Lipocortin I and II expression* in vivo*:* Immunoperoxidase staining of bronchi sections ($n = 3$) with anti-lipocortin I and II antibodies showed a strong expression in the bronchial epithelium (Fig. 1). Similar staining patterns were observed for lipocortin I and II. Positive staining for lipocortin I and II was also found in the epithelial cells of the submucosal glands.

*Lipocortin I and II expression* in vitro*:* In cultured HBEC and in BEAS 2B cells lipocortin expression was studied using FACScan and Northern blot analysis. With FACScan analysis we found that more than 99% of BEAS 2B cells were positive for both intracellular lipocortin I and II (Fig. 2). Fifty and 80% of cultured HBEC ($n = 3$) were positive for intracellular lipocortin I and II, respectively. A representative experiment is shown in Fig. 2. Northern blot analysis showed expression of lipocortin I and II mRNA, 1.4 and 1.6 kb respectively, in both cultured HBEC and BEAS 2B cells (Figs 3A and 3B).

*Effects of IL-1β,* TNF-α, *LPS and dexamethasone on lipocortin I and II mRNA and protein*

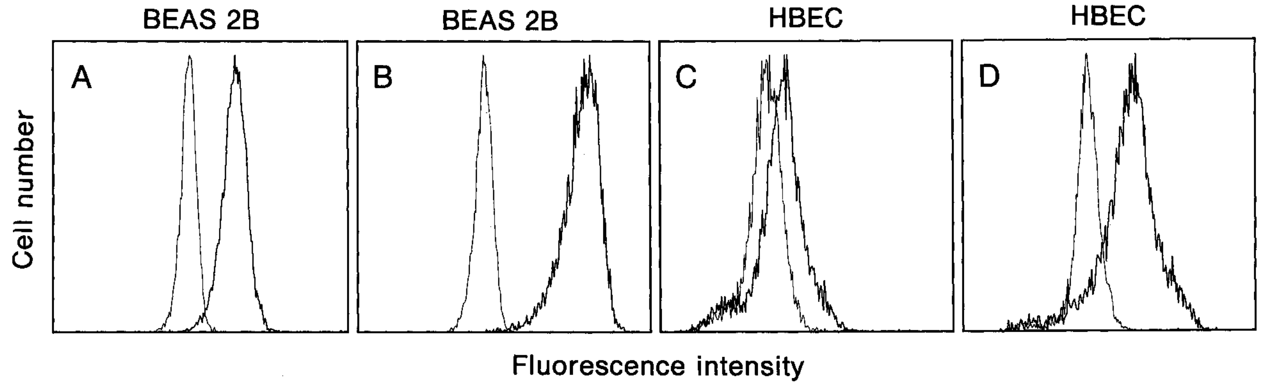

FIG. 2. FACS analysis of intracellular lipocortin I (A,C) and II (B,D) expressions in BEAS 2B cells and in HBEC. Representative histograms, indicating nonspecific fluorescence intensity (thin line) and lipocortin I or II expression (bold line) are shown.

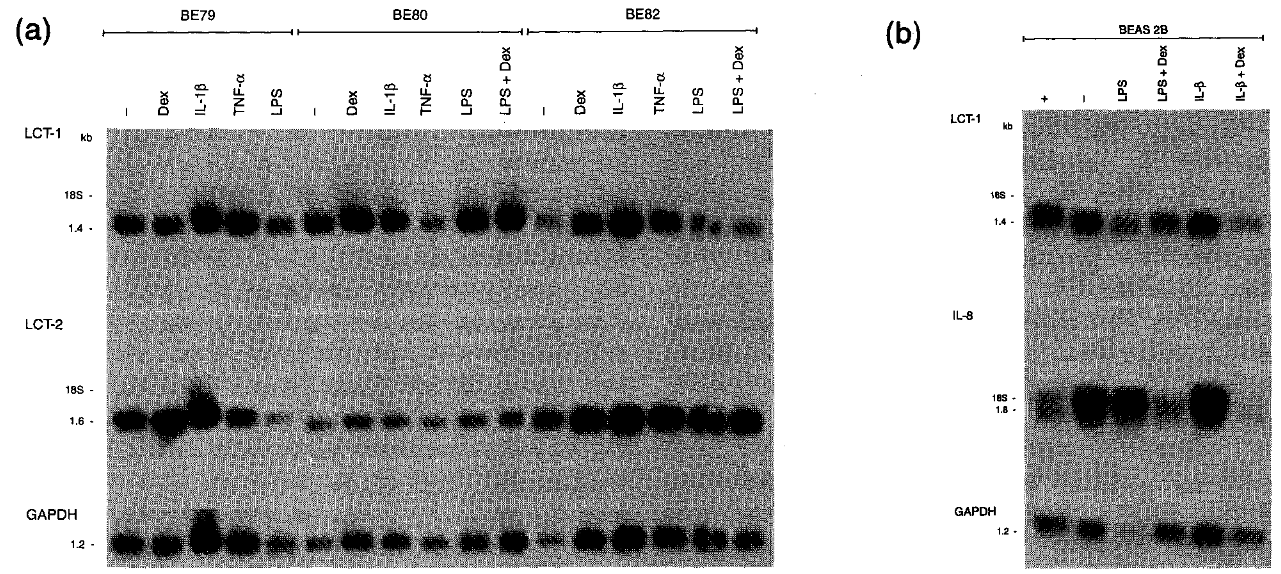

FIG. 3. Effects of IL-1β, TNF-α, LPS and dexamethasone on lipocortin I and II mRNA expression. Northern blot analysis of HBEC (A) and BEAS 2B cells (B). HBEC from three different patients were cultured for 24 h in a basal medium of DMEM-/F12 (lanes 1,6,12), with $10^{-6}$ M dexamethasone (lanes 2,7,13), with 20 ng/ml IL-1β (lanes 3,8,14), with 20 ng/ml TNF-α (lanes 4,9,15), with 100 μg/ml LPS (lanes 5,10,16), or with 100 μg/ml LPS and $10^{-6}$ M dexamethasone (lanes 11,17). BEAS 2B cells were cultured for 24 h in DMEM/F12 supplemented with insulin, hydrocortisone, transferrin, EGF and FCS (lane 1), in a basal medium of DMEM/F12 (lane 2), with 10 μg/ml LPS (lane 3), with 10 μg/ml LPS and $10^{-6}$ M dexamethasone (lane 4), with 20 ng/ml IL-1β (lane 5), or with 20 ng/ml IL-1β and $10^{-6}$ M dexamethasone (lane 6). The filters were hybridized to $^{32}$P-labelled lipocortin I (LCT-1), lipocortin II (LCT-2), IL-8 and GAPDH probes.

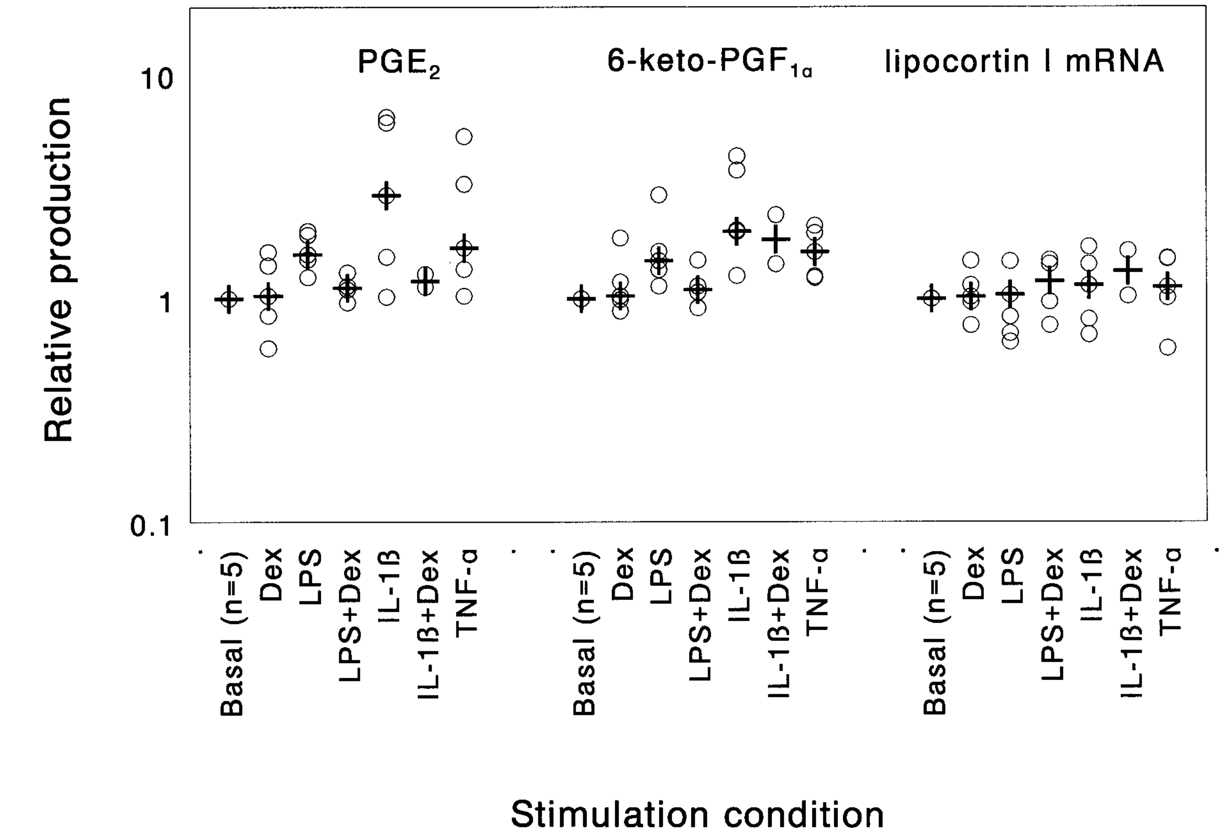

FIG. 4. Effect of 24 h incubation with IL-1β (20 ng/ml), TNF-α (20 ng/ml), LPS (100 μg/ml) and dexamethasone ($10^{-6}$ M) on the basal lipocortin I mRNA expression and the basal release of $PGE_2$ and 6-keto-$PGF_{1\alpha}$ in cultures of HBEC ($n$ = 5). $PGE_2$ and 6-keto-$PGF_{1\alpha}$ release and lipocortin I mRNA expression are expressed relative to the basal production. Lipocortin I mRNA expression was corrected for GAPDH mRNA expression. Median values are represented by a (+).

*expression:* To investigate the effect of inflammatory age and dexamethasone on lipocortin I and II mRNA and protein expression, HBEC and BEAS 2B cells were incubated with different concentrations of IL-1β, TNF-α, LPS and dexamethasone. Incubation for 24 h with IL-1β, TNF-α, LPS or dexamethasone did not significantly affect lipocortin I or II mRNA expression in cultured HBEC or in BEAS 2B cells (Figs 3A and 3B). IL-8 mRNA was used as a positive control, as it has been shown previously to increase upon stimulation with IL-1β or LPS, and to decrease upon incubation with dexamethasone. Figure 3B shows that IL-8 mRNA expression in BEAS 2B cells was decreased after incubation with dexamethasone, whereas lipocortin I mRNA expression was not increased. The results of the experiments ($n$ = 5), in which the effects of IL-1β, TNF-α, LPS and dexamethasone on the basal lipocortin I mRNA expression in cultures of HBEC were studied, is shown in Fig. 4. Using FACScan analysis and ELISA no effect of IL-1β, TNF-α, LPS or dexamethasone was observed on the lipocortin I or II protein expression in HBEC or BEAS 2B cells (data not shown).

*Effects of IL-1β, TNF-α, LPS and dexamethasone on $PGE_2$ and 6-keto-$PGF_{1\alpha}$ production, and correlation with lipocortin mRNA expression:* The effect of IL-1β, TNF-α, and LPS on $PGE_2$ and 6-keto-$PGF_{1\alpha}$ production was measured, and we studied whether the expected inhibition of stimulated eicosanoid production by dexamethasone correlated with an induction of lipocortin I and II. Incubation of HBEC ($n$ = 5) for 24 h with either IL-1β, or TNF-α or LPS significantly increased basal $PGE_2$ and 6-keto-$PGF_{1\alpha}$ production ($p$ < 0.01) (Fig. 4). $PGE_2$ and 6-keto-$PGF_{1\alpha}$ production in one representative culture of HBEC is shown in Table 1. The LPS- and IL-1β-mediated increases in $PGE_2$ and 6-keto-$PGF_{1\alpha}$ production were reduced by dexamethasone. Reduction of the LPS-mediated increase of $PGE_2$ by dexamethasone was statistically significant ($p$ < 0.05). Dexamethasone also clearly reduced the IL-1β-mediated increase of both $PGE_2$ and 6-keto-$PGF_{1\alpha}$, but statistical significance could not be reached because of low sample number. Reduction of the IL-1β- and LPS-mediated $PGE_2$ and 6-keto-$PGF_{1\alpha}$ production by dexamethasone was not accompanied by an increase in lipocortin I

**Table 1.** $PGE_2$ and 6-keto-$PGF_{1\alpha}$ production in a representative culture of HBEC

| Stimulation condition[a] | $PGE_2$[b] | 6-keto-$PGF_{1\alpha}$[b] |
|---|---|---|
| Basal | 5.68 | 1.60 |
| Dex | 3.43 | 1.42 |
| LPS | 8.98 | 4.68 |
| LPS + Dex | 7.50 | 1.45 |
| IL-1β | 16.51 | 3.25 |
| IL-1β+Dex | 7.31 | 2.29 |
| TNF-α | 7.71 | 2.60 |

[a]HBEC were incubated for 24 h with IL-1β (20 ng/ml), TNF-α (20 ng/ml), LPS (100 μg/ml) and dexamethasone (Dex) ($10^{-6}$ M).

[b]$PGE_2$ and 6-keto-$PGF_{1\alpha}$ production are expressed as $ng.ml^{-1}.mg^{-1}$ total RNA.

mRNA (Fig. 4) or lipocortin II mRNA (data not shown).

*Analysis of the inducibility of lipocortin I and II mRNA by dexamethasone:* To evaluate the effect of dexamethasone on lipocortin I and II mRNA expression more precisely we cultured HBEC and BEAS 2B cells for up to 5 days without glucocorticoids. The cells were then incubated with different concentrations of dexamethasone ($10^{-10}$, $10^{-9}$, $10^{-8}$, $10^{-7}$, and $10^{-6}$ M) and with $10^{-6}$ M dexamethasone for various lengths of times (2, 4, 6, 10, 24 and 48 h). Both the effects of dexamethasone on cells cultured in a medium of DMEM/F12 without supplements and on cells in a medium of DMEM/F12 supplemented with insulin, transferrin, EGF, $Na_2SeO_3$, and charcoal-stripped FCS, but without hydrocortisone were examined. Under none of these conditions dexamethasone affected the expression of lipocortin I or II mRNA (data not shown).

### Discussion

We demonstrated in this study that lipocortin I and II are expressed in cultured HBEC, a bronchial epithelial cell line and in the epithelium in bronchi sections. Immunoperoxidase stainings of human bronchi sections with anti-lipocortin I and II antibodies showed a clear expression in the bronchial epithelium and in the epithelial cells of the submucosal glands. In cultured HBEC and in the BEAS 2B cell line, we demonstrated the presence of lipocortin I and II mRNA and protein by Northern blotting and FACScan analysis, respectively. Lipocortin I and II mRNA and protein expression in the HBEC was not affected by incubation of the cells with IL-1β, TNF-α, LPS or dexamethasone. The HBEC were able to respond to these stimuli, as the production of $PGE_2$ and 6-keto-$PGF_{1\alpha}$ significantly increased upon incubation with IL-1β, TNF-α or LPS. This increased production was reduced by dexamethasone. Reduction by dexamethasone of the stimulated production of $PGE_2$ and 6-keto-$PGF_{1\alpha}$ was not accompanied by an increase in lipocortin I or II expression. Thus, in HBEC this particular glucocorticoid action is not mediated through lipocortin I or II induction.

Our findings on the expression of lipocortin I in the human bronchus are in agreement with those in the airway epithelium of the rat, studied by Fava *et al.*[29] They demonstrated the presence of lipocortin I in the rat epithelium of nasal, tracheal and bronchial airways, and in the ductal epithelium of various glands. Production of lipocortin-like proteins in cultured human tracheal submucosal gland cells has been described by Jacquot *et al.*[19] In the foetal and neonatal human lung, lipocortin I immunostaining was found in the bronchiolar epithelium as early as 12 weeks, beginning with the largest airways, and by 24 weeks extending distally to the bronchioloalveolar portals.[30] In bovine bronchial epithelial cells a differential expression of lipocortin I and II was found.[16] Lipocortin I was expressed in the ciliated cells, whereas lipocortin II was expressed in the basal cells. We did not find such a differential expression of lipocortin I and II in human bronchi in our studies.

The bronchial epithelium is considered to play an important role in inflammatory and immunological reactions by production of a variety of inflammatory mediators as can be observed in inflammatory pulmonary diseases such as asthma. In addition to this role, the bronchial epithelium could also be involved in anti-inflammatory reactions through the production of anti-inflammatory proteins, e.g. lipocortins. In this study we found a high expression of lipocortin I and II in the human bronchial epithelium. A physiological role has been proposed for lipocortin I recently. Lipocortin I may function as a 'barrier' to inappropriate inflammatory and autoimmune responses at specific sites around the body.[7] Following an inflammatory stimulus, stress-induced stimulation of the hypothalamic–pituitary–adrenal axis (HPA) axis may result in the release of cortisol, which in turn leads to the production of lipocortin I. The lipocortin-binding molecules on monocytes and neutrophils arriving at tissue sites are expected to become saturated, with resultant moderation of migratory and/or pro-inflammatory activities. Others have speculated from results in animal studies that epithelial cell damage and loss, as seen in asthmatics, could decrease availability of these protective properties of lipocortin I.[16] Increased inflammation would thereby be predicted.

We did not observe an induction of lipocortin

I or II by dexamethasone in cultures of HBEC or in the BEAS 2B cell line. An elevated production of lipocortin I in response to glucocorticoids has been demonstrated in human peripheral blood monocytes, human alveolar macrophages, rat alveolar epithelial cells and in bovine bronchial epithelial cells.[15–18] Glucocorticoids increased amounts of lipocortin I in these cells in a dose dependent manner. However, Brönnegard *et al.* did not observe an induction of lipocortin I mRNA by dexamethasone in seven different cell types, including primary human macrophages.[11] The synthesis of lipocortin I does not appear to be under glucocorticoid control in certain cell lines and has been linked with cell differentiation events in some cases.[7,31,32] Isacke *et al.* were unable to observe any effect of dexamethasone treatment in U-937 cells under a variety of conditions on the expression of lipocortin I or II, nor did dexamethasone induce their secretion.[10] An induction by dexamethasone of the expression of mRNA of lipocortin I and II and the release of lipocortin I and V was observed in differentiated, but not in undifferentiated U-937 cells by Sollito *et al.*[32]

For the induction experiments we used both HBEC, just a few days after isolation and after culturing for 1 or 2 weeks. Other studies point to the importance of culture conditions in studying synthesis of lipocortins by cultured cells.[31] In our experiments we have used charcoal-stripped FCS in the culture medium so that endogenous steroids, which might influence lipocortin synthesis, are removed. We have incubated epithelial cells for up to 5 days in steroid-free medium prior to the addition of dexamethasone, to ensure that the cells were in an unstimulated condition at the beginning of the experiment. As growth factors, such as EGF, are thought to induce lipocortin synthesis, we have also performed experiments, in which cells were cultured in a basal medium without growth factors. In all cases we found a clear mRNA and protein expression of lipocortin I and II in HBEC, but no induction of lipocortins by dexamethasone after incubation for various lengths of times with different concentrations. After culturing for 5 days in steroid free medium, lipocortin I mRNA was expressed constitutively at approximately 90% of the lipocortin I mRNA level in complete medium, containing hydrocortisone. Vishwanatha *et al.* found in bovine bronchial epithelial cells, that the constitutive expression of lipocortin I was 20% of the lipocortin I level in medium containing hydrocortisone after culturing for 5 days.[16] These studies indicate that there is a difference between species in the inducibility of lipocortins by glucocorticoids.

In summary, we found a high expression of lipocortin I and II in the bronchial epithelium compared to the underlying layers in human bronchi sections and demonstrated that glucocorticoids do not increase the expression of lipocortin I and II in HBEC. Our study indicates, that decrease in $PGE_2$ and 6-keto-$PGF_{1\alpha}$ production in HBEC upon incubation with glucocorticoids is not mediated by increased expression of lipocortins.

### References

1. Marini M, Soloperto M, Mezzetti M, Fasoli A, Mattoli S. Interleukin-1 binds to specific receptors on human bronchial epithelial cells and upregulates granulocyte/macrophage colony-stimulating factor synthesis and release. *Am J Respir Cell Mol Biol* 1991; **4**: 519–524.
2. Cromwell O, Hamid Q, Corrigan CJ, *et al* Expression and generation of interleukin-8, IL-6 and granulocyte-macrophage colony-stimulating factor by bronchial epithelial cells and enhancement by IL-1β and tumour necrosis factor-α. *Immunol* 1992; **77**: 330–337.
3. Becker S, Quay J, Koren HS, Haskill JS. Constitutive and stimulated MCP-1, GROα, β, and gamma expression in human airway epithelium and bronchoalveolar macrophages. *Am J Physiol* 1994; **266**: L278–L286.
4. Mattoli S, Masiero M, Calabrò F, Mezzetti M, Plebani M, Allegra L. Eicosanoid release from human bronchial epithelial cells upon exposure to toluene diisocyanate *in vitro*. *J Cell Physiol* 1990; **142**: 379–385.
5. Salari H, Chan-Yeung M. Release of 15-hydroxyeicosatetraenoic acid (15-HETE) and prostaglandin $E_2$ ($PGE_2$) by cultured human bronchial epithelial cells. *Am J Respir Cell Mol Biol* 1989; **1**: 245–280.
6. Wallner BP, Mattaliano RJ, Hession C, *et al.* Cloning and expression of human lipocortin, a phospholipase $A_2$ inhibitor with potential anti-inflammatory activity. *Nature* 1986; **320**: 77–81.
7. Goulding NJ, Guyre PM. Regulation of inflammation by lipocortin I. *Immunol Today* 1992; **13**: 295–297.
8. Flower RJ. Lipocortin and the mechanism of action of the glucocorticoids. *Br J Pharmacol* 1988; **94**: 987–1015.
9. Piltch A, Sun L, Fava RA, Hayashi J. Lipocortin-independent effect of dexamethasone on phospholipase activity in a thymic epithelial cell line. *Biochem J* 1989; **261**: 395–400.
10. Isacke CM, Lindberg RA, Hunter T. Synthesis of p36 and p35 is increased when U-937 cells differentiate in culture but expression is not inducible by glucocorticoids. *Mol Cell Biol* 1989; **9**: 232–240.
11. Brönnegard M, Andersson O, Edwall D, Lund J, Norstedt G, Carlstedt-Duke J. Human calpactin II (lipocortin I) messenger ribonucleic acid is not induced by glucocorticoids. *Mol Endocrinol* 1988; **2**: 732–739.
12. Flower RJ, Rothwell NJ. Lipocortin-1: cellular mechanisms and clinical relevance. *TIPS* 1994; **15**: 71–76.
13. Pepinsky RB, Sinclair LK, Browning JL, *et al.* Purification and partial sequence analysis of a 37-kDa protein that inhibits phospholipase $A_2$ from rat peritoneal exudates. *J Biol Chem* 1986; **261**: 4239–4246.
14. Peninsky BB, Tizard R, Mattaliano RJ, *et al.* Five distinct calcium and phospholipid binding proteins share homology with lipocortin I. *J Biol Chem* 1988; **263**: 10799–10811.
15. Ambrose MP, Hunninghake GW. Hunninghake GW. Corticosteroids increase lipocortin I in alveolar epithelial cells. *Am J Respir Cell Mol Biol* 1990; **3**: 349–353.
16. Vishwanatha JK, Muns G, Beckmann JD, Davis RG, Rubenstein I. Differential expression of annexins I and II in bovine bronchial epithelial cells. *Am J Respir Cell Mol Biol* 1995; **12**: 280–286.
17. Ambrose MP, Bahns CLC, Hunninghake GW. Lipocortin-1 production by human alveolar macrophages. *Am J Resp Cell Mol Biol* 1992; **6**: 17–21.
18. Caterina de R, Sicari R, Giannessi D, *et al.* Macrophage-specific eicosanoid synthesis inhibition and lipocortin-1 induction by glucocorticoids. *J Appl Physiol* 1993; **75**: 2368–2375.
19. Jacquot J, Dupuit F, Elbtaouri H, *et al.* Production of lipocortin-like proteins by cultured human tracheal submucosal gland cells. *FEBS Lett* 1990; **274**: 131–135.
20. Reddel RR, Ke Y, Gerwin BI, *et al.* Transformation of human bronchial epithelial cells by infection with SV40 or adenovirus-12 SV40 hybrid virus or transfection via strontium phosphate coprecipitation with a plasmid containing SV40 early region genes. *Cancer Res* 1988; **48**: 1904–1909.
21. Albright CD, Grimley PM, Jones RT, Fontana JA, Keenan KP, Resau JH. Cell-to-cell communication: a differential response to TGF-β in normal and transformed (BEAS-2B) human bronchial epithelial cells. *Carcinogenesis* 1991; **12**: 1993–1999.
22. Lechner JF, Haugen A, McClendon IA, Pettis EW. Clonal growth of normal adult human bronchial cells in a serum-free medium. *In Vitro* 1982; **18**: 633–642.

23. Chomczynski P, Sacchi M. Single step method of RNA isolation by thiocyanate–phenol–chloroform extraction. *Annal Biochem* 1987; **162**: 156–162.
24. Versnel MA, Bouts MJ, Langerak AW, *et al.* Hydrocortisone-induced increase of PDGF β-receptor expression in a human malignant mesothelioma cell line. *Exp Cell Res* 1992; **200**: 83–88.
25. Benham FJ, Hodgkinson S, Davies KE. A glyceraldehyde-3-phosphate dehydrogenase pseudogene on the short arm of the human X-chromosome defines a multigene family. *EMBO J* 1984; **3**: 2635–2640.
26. Koning H, Baert MRM, de Groot R, Neijens HJ, Savelkoul HFJ. Analysis of cytokine gene expression in stimulated T cells of small children by semi-quantitive PCR. *Med Inflam* 1995; **4**: 196–204.
27. Smith T, Smith SF, Buckingham JC. A competitive ELISA for the estimation of rat lipocortin I. *Biochem Soc Trans* 1990; **18**: 1230–1231.
28. Zijlstra F, Vincent JE, Mol WM, Hoogsteden HC, van Hal PThW, Jongejan RC. Eiocosanoid levels in bronchoalveolar lavage fluid of young female smokers and non-smokers. *Eur J Clin Invest* 1992; **22**: 301–306.
29. Fava RA, Mckanna J, Cohen S. Lipocortin I (p35) is abundant in a restricted number of differentiated cell types in adult organs. *J Cell Physiol* 1989; **141**: 284–293.
30. Johnson MD, Gray ME, Carpenter G, Pepinsky RB, Stahlman MT. Ontogeny of epidermal growth factor receptor and lipocortin-1 in fetal and neonatal human lungs. *Hum Pathol* 1990; **21**: 182–191.
31. Croxtall JD, FLower RJ. Lipocortin 1 mediates dexamethasone-induced growth arrest of the A549 lung adenocarcinoma cell line. *Proc Natl Acad Sci* 1992; **89**: 3571–3575.
32. Solito E, Raugei G, Melli M, Parente L. Dexamethasone induces the expression of mRNA of lipocortin 1 and 2 and the release of lipocortin 1 and 5 in differentiated but not undifferentiated U937 cells. *FEBS Lett* 1991; **291**: 238–244.

ACKNOWLEDGEMENTS. We gratefully acknowledge Professor Dr R. Benner for his continuous support. We thank Dr S. F. Smith for measuring extracellular lipocortin I levels. Dr J. Lechner kindly provided the BEAS 2B cell line, Mr T. M. van Os is acknowledged for excellent photographic assistance. Dr H. Koning for computer software development and Ms P. C. Assems for secretarial assistance. This study was supported by the Netherlands Asthma Foundation (90.44).

**Received 26 February 1996; accepted 14 March 1996**
